# Correlation between Male Social Status, Testosterone Levels, and Parasitism in a Dimorphic Polygynous Mammal

**DOI:** 10.1371/journal.pone.0012507

**Published:** 2010-09-13

**Authors:** Sandra S. Negro, Abigail K. Caudron, Michel Dubois, Philippe Delahaut, Neil J. Gemmell

**Affiliations:** 1 School of Biological Sciences, University of Canterbury, Christchurch, New Zealand; 2 BiopTis s.a., Université de Liège, Sart Tilman, Belgium; 3 Institut de Zoologie, Université de Liège, Liège, Belgium; 4 Hormonologie, Centre d'Economie Rurale, Marloie, Belgium; 5 Department of Anatomy and Structural Biology, University of Otago, Dunedin, New Zealand; Stanford University, United States of America

## Abstract

Life history trade-offs have often been assumed to be the consequence of restrictions in the availability of critical resources such as energy and nutrients, which necessitate the differential allocation of resources to costly traits. Here, we examined endocrine (testosterone) and health (parasite burdens) parameters in territorial and non-territorial New Zealand fur seal males. We documented intra-sexual differences in sexual behaviours, testosterone levels, and parasitism that suggest a trade-off exists between reproductive success and physical health, particularly susceptibility to helminths and acanthocephalans, in males displaying different mating tactics (i.e., territorial and non-territorial tactics). Levels of testosterone were higher in territorial males and correlated positively with reproductive effort (i.e., intra- and inter-sexual interactions). However, these territorial males also exhibited high levels of parasitic infection, which may impair survival in the long-term. Our study, while limited in sample size, provides preliminary evidence for a link between male mating tactics, testosterone levels and parasite loads, and potential effects on reproductive success and life history that should be explored further.

## Introduction

Life history trade-offs have often been assumed to be the consequences of restrictions in the availability of critical resources such as energy and nutrients, necessitating differential allocation of resources to costly traits [1]. Increasing evidence implicates hormones as mediators of life history trade-offs [1], [2]. Hormones transduce environmental cues and regulate transitions between life-cycle stages (e.g. metamorphosis, maturation, and reproduction) in which organisms face developmental constraints [3]. Importantly, hormones can have pleiotropic and often antagonistic effects on morphological, physiological and behavioural characters [4]. Testosterone, the principal androgenic hormone, can raise male mating success by promoting the development of secondary sexual characters. Testosterone promotes aggressive behaviours associated with courtship and reproduction in vertebrates [5], [6], [7], while decreasing fitness at the same time by impairing traits such as parental care and immune system [8], [9]. Testosterone interacts with the immune system at the level of both individual cells involved in the humoral- and cellular-mediated immunity, and glands or tissues implicated in immune functions [10]. A schematic model of interactions between endocrine system, immune system, secondary sexual characters and parasites has previously been proposed in Folstad and Karter [9]. Testosterone may have a negative impact on the immune system when beyond a threshold level [11], [12]. Wingfield et al. [8] proposed a 3-level model of testosterone in male birds: a non-breeding androgen baseline (level A), the breeding androgen baseline induced by environmental cues (level B), and the physiological testosterone maximum that an individual can achieve during intra- and inter-sexual interactions (level C). Level B is necessary for spermatogenesis, expression of sexual behaviour, and the appearance of some secondary sexual characters, whereas level C is facultative and triggered by social stimuli or challenge during the breeding season. Level C corresponds to the frequency and intensity of territorial aggressiveness and female defence behaviour [8]. The above models described for birds are supported by other studies in pinnipeds [13], [14], [15].

New Zealand fur seals *Arctocephalus forsteri* (Lesson 1828) are polygynous and annual colonial breeders. *Arctocephalus forsteri* displays moderate polygyny where one male may mate with 4–15 females over the course of a single breeding season [16], [17]. The breeding season begins when adult males come ashore and establish territories in the austral spring. Females haul out from mid-November to late December to give birth [18]. Females enter oestrus approximately one week after parturition followed by alternation between foraging at sea and pup-nursing onshore. The breeding season ends when all females terminate their oestrous cycle and initiate their foraging cycles in February [18]. The life history traits of *A. forsteri* and other pinniped species are linked by several fundamental characteristics related to the evolution of their socio-sexual behaviour. They are marine feeders, but come onshore for parturition and postnatal pup care [19]. Reproduction in pinnipeds is characterised by highly synchronised pupping and mating (except for the tropical and subtropical species of seal such as Galapagos fur seals and monk seals which have a lower degree of synchrony), in addition to a delayed implantation of the blastocyst [20], [21]. Australian sea lions which have an asynchronous 18 month breeding cycle between colonies is the extreme exception to this common pattern [22].

In Mammals, little is known about the intra-sexual differences in physiology and the impacts of parasite infections on the reproductive success of male of different social status, employing different mating tactics [23], [24], [25], [26]. This study examined endocrine (testosterone) and health (parasite loads) parameters in *A. forsteri* males employing the territorial mating tactic and non-territorial mating tactics related to their relative reproductive success. Our first aim was to collect behavioural observations on individually identified males which allowed us to assign these males a mating tactic. The second aim was to measure testosterone levels in males displaying territorial and non-territorial mating tactics using non-invasive sampling methods. The third aim was to examine the parasitic load and species diversity in relation to the social status of the study males. Finally, we correlated the above results with the relative reproductive success of the territorial and non-territorial male mating tactics determined in a parallel study investigating the same focal area [17].

## Materials and Methods

### Study site

The Ohau Point seal breeding colony, ca. 2,200 individuals [27], located 26km north of the Kaikoura township, New Zealand (42°25′0″S, 173°40′60″E), is a narrow rookery colony (50–100m wide) that runs 1km along the Pacific Ocean coast and is situated close to fur seal foraging ground**s** [27]. Our study area was limited to the central portion of the colony (50–90m wide and 120m long, backed by a cliff on the west). A permit (Per/10/2002/01) to undertake marine mammal research (Marine Mammal Protection Act 1978) was given by the New Zealand Department of Conservation.

### Behavioural sampling

We undertook 223 hours of field observations spread from mid-November 2004 to early January 2005 coinciding with the arrival of pregnant females and covering the period of highest intra- and inter-sexual interactions. Fifty-two males that spent more than one hour during the study period were readily identifiable by natural marking (e.g. body scars, flipper irregularities) or artificial marking (i.e. cattle ear tag or sheep tag from previous studies) and these were selected for further study. Three out of fifty two identified breeding males spent less than three hours at our study site and were excluded from the analyses. We recorded all interactions involving focal males by “all occurrences sampling” [28]. We categorised these interactions as described in Caudron et al. [17] with the following modifications. The intra-sexual interactions involved walking towards, following, chasing, attacking, lunging at, and fighting with another male. We attributed vocalizations to either apparently addressed to a male target or non-addressed. For each intra-sexual interaction, the study males were classified as aggressive (the male initiating the interaction and/or displacing or triumphing over the other male) or submissive (the target male and/or displaced male). The inter-sexual interactions involved walking towards, withholding, attempting to copulate and successful copulation, accepting or tolerating female advances (soliciting, mounting on the male's back, and biting the male's neck) and vocalizing to a female.

We used seven variables described in detail in Caudron et al. [17], to quantify the breeding behaviour of the study males: 1) the occurrence of aggressive intra-sexual interactions, 2) the occurrence of submissive intra-sexual interactions, 3) the occurrence of inter-sexual interactions, 4) the occurence of male vocalization to female, 5) the occurrence of male vocalization to addressed male target or non-addressed, 6) defence or non-defence of territory hosting females, and 7) the total number of days spent on the site. The number of copulations that could be observed during the study breeding season was very low. Previous studies in fur seals and several other pinnipeds have identified discrepancies between behavioural (i.e. copulations) and genetic (i.e. paternity) measurements of male reproductive success [29], [30]. We think that the number of copulations is not a reliable measure of male reproductive success. It was not possible to use male size and male reproductive success from Caudron et al. [17] because we had only four males from our study that were studied in Caudron et al. [17].

### Testosterone assays

We used a non-invasive sampling approach to collect faecal (n = 10) and urine (n = 7) sample sizes for the testosterone assays from different individual males in our study area, for which extensive behavioural data were available. Our sample sizes were small because during the breeding season males fast and excrete at a low rate, and samples are only useful if fresh, thus sampling opportunities are understandably scarce. For reference samples, we also collected one faecal sample and several urine samples from females. The sample collections spread from mid-November 2004 to early January 2005 from the periphery or outside the male territories to minimise disturbance. This period corresponds to the period of highest intra- and inter-sexual interactions [17]. Territorial males generally urinated or defecated after chasing a subordinate male or on their way back to the centre of their territory. Samples were collected at the time of excretion with a sterile spoon or syringe from the rocky surface. Faecal and urine samples for the testosterone assays were stored at −20°C until assayed (ca. 3–4 months later).

We performed a preliminary extraction step [31], [32] followed by a purification step using immunoaffinity chromatography columns and reverse-phase high performance liquid chromatography prior to the testosterone radioimmunoassay. A detailed protocol of the testosterone assays is described in Negro [33]. The intra-assay coefficients of variation (i.e. variation of testosterone assays estimated from measuring all samples in duplicate in a same run) in urine and faeces were 20% and 25%, respectively. The limit of detection was 0.2 ng/ml in urine and 0.3 ng/g dry faeces for the faecal samples. Extraction efficiency was determined by the recovery of ^3^H-Testosterone (6,000 cpm) added to the samples prior to extraction and was 33±5% (mean±SD) for urine and 18±3% for faeces.

### Parasitic load and species richness in faecal samples

We used the same faecal samples (n = 10) collected for the testosterone assays in addition to faecal samples collected from other known males (n = 2) to estimate the parasitic load and species diversity (trematodes, cestodes, nematodes, acanthocephalans). A modified McMaster quantitative method was used to estimate the number of parasite eggs per gram of faeces [34]. We added 56ml of saturated NaCl (density of 1200 kg.m^−3^ at 21°C) to 4 g of faecal material. After mixing, we filtered the solution through a 150µm sieve. We took a few ml of the suspension and filled in the two chambers (10×10 mm) of the McMaster slide and let the suspension rest for 5min before observing the slide with a microscope at 10×10 magnification. The egg count for each parasitic species was multiplied by 25 to determine the number of eggs per gram of faeces.

### Statistical analyses

Parametric statistical methods (Student's and Welch's Two Sample *t*-tests) were used except when the assumption of normality (Shapiro Wilk test) of the data distribution was violated. In this situation, nonparametric tests (Mann-Whitney U Test, Spearman rank correlations) were used. For identifying male behavioural classes, we used an agglomerative hierarchical clustering based on similarities in their behavioural profile using Ward's linkage of Euclidean distances [35], [36]. The significance of the difference in testosterone levels in territorial and non-territorial males were tested by randomisation test (resampling based test). This robust statistical test was used because of the small sample sizes. The probability level used for significance was α = 0.05. Statistical analyses were performed in STATISTICA v8 [36] and R v1.7.1 [37].

## Results

### Behavioural sampling

**Figure 1:**
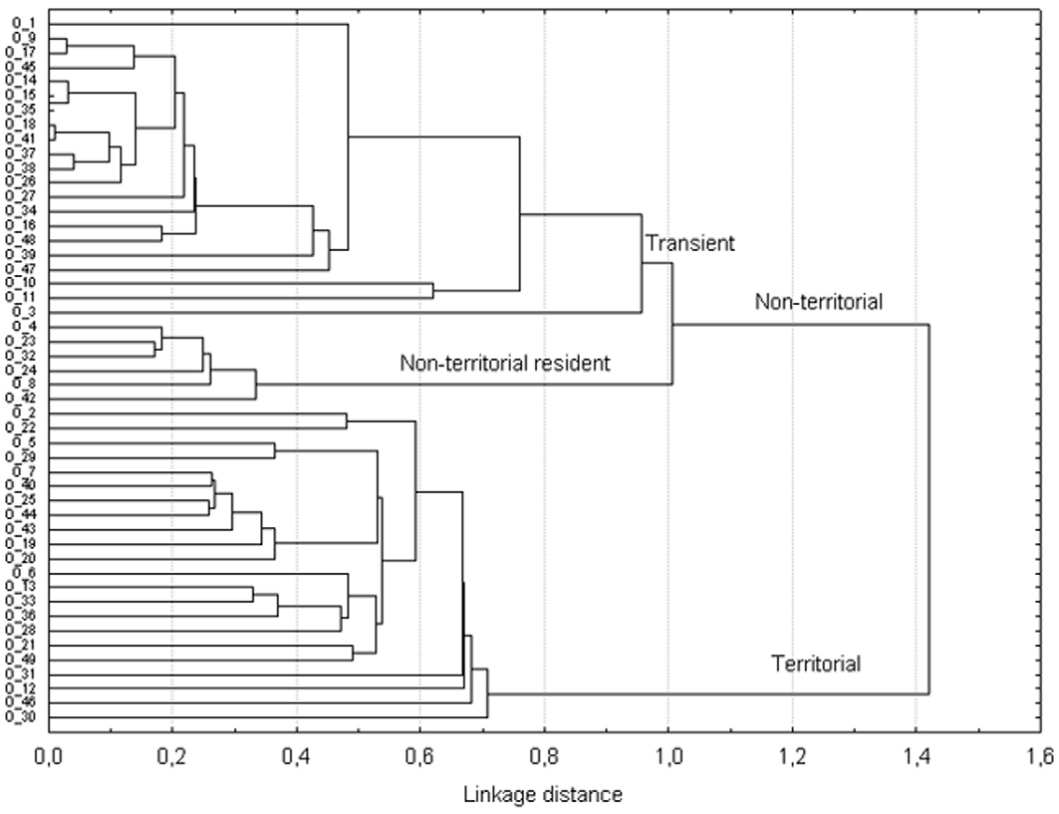
Hierarchical clustering of male behavioural profiles using Ward's linkage of Euclidean distances. The method builds the hierarchy from the individual elements by progressively merging clusters. The amount of clustering structure that has been found for each node is interpreted by the Agglomerative Coefficient (0.9759) (http://www.wessa.net/rwasp_agglomerativehierarchicalclustering.wasp/). The y-axis represents the male ID.

We used seven variables to describe the breeding behaviours of the 49 focal males. The cluster analysis identified two classes, the territorial profile and the non-territorial profile (Figure 1). Two sub-classes are observed within the non-territorial class. One sub-class corresponds to non-territorial visitors wherein these transient males spent a few days (mean±SE; 2.19±0.33) on the site, but at various locations. The other sub-class corresponds to non-territorial residents wherein these resident males spent more days (12.50±2.36) on the site than the transient males and showed breeding site-fidelity [38], [39]. Caudron et al. [17] qualify males in these sub-classes as using alternative mating tactics. The descriptive statistics of behavioural observations are summarized in Table 1. We identified 12 topographically demarcated territories defended by territorial males.

**Table 1:** Descriptive statistics of behavioural observations for the territorial, non-territorial transient and resident male categories.

	male/male interactions per hour			male/female interactions per hour		Days on site
	aggressive	submissive	call to male/unaddressed	Reproductive behaviour	call to female	
	**Territorial (n = 22)**					
**mean**	0.63	0.07	0.44	1.06	0.35	21.23
**median**	0.51	0.06	0.40	1.05	0.25	16.50
**SE**	0.09	0.01	0.07	0.09	0.06	2.4
**SD**	0.41	0.07	0.33	0.45	0.30	11.27
**95% CI**	0.449–0.820	0.037–0.097	0.296–0.586	0.862–1.259	0.216–0.484	
	**Non-territorial transient (n = 21)**					
**mean**	0.09	0.20	0.11	0.32	0.03	2.19
**median**	0.00	0.19	0.00	0.28	0.00	2.00
**SE**	0.04	0.03	0.06	0.09	0.03	0.33
**SD**	0.18	0.16	0.28	0.41	0.12	1.54
**95% CI**	0.006–0.169	0.121–0.271	0–0.235	0.135–0.514	0–0.083	
	**Non-territorial resident (n = 6)**					
**mean**	0.18	0.27	0.05	0.29	0.09	12.50
**median**	0.19	0.29	0.03	0.27	0.09	10.50
**SE**	0.03	0.06	0.02	0.08	0.02	2.36
**SD**	0.08	0.14	0.06	0.19	0.05	5.79
**95% CI**	0.097–0.268	0.119–0.423	0–0.115	0.089–0.483	0.029–0.146	

Territorial males showed a higher mean frequency of aggressive intra-sexual interactions (Mann-Whitney U Test; territorial vs resident, U = 5, p = 0.0001; territorial vs transient, U = 33, p<0.0001), a high frequency of male-female interactions (territorial vs resident, U = 5, p = 0.0001; territorial vs transient, U = 42, p<0.0001) and of vocalizations addressed and non-addressed (territorial vs resident, U = 10, p = 0.0007; territorial vs transient, U = 56, p<0.0001) compared to the non-territorial males (Table 1). Territorial males also showed a lower frequency of submissive intra-sexual interactions (territorial vs resident, U = 16, p = 0.0034; territorial vs transient, U = 130, p = 0.0146). Finally, the territorial males vocalised to females at a higher rate compared to non-territorial males (territorial vs resident, U = 27, p = 0.0283; territorial vs transient, U = 17, p<0.0001) (Table 1).

### Testosterone assays

Faecal testosterone was significantly higher for territorial males compared to non-territorial males (Welch's two sample *t*-test, t = 4.94, df = 3.21, p<0.01) (Table 2). Concerning urine, testosterone levels in territorial males were high compared to non-territorial males, but the statistical test was not worth conducting with the low number of territorial male samples (n = 2). Female testosterone levels in faeces (2.1 ng/g dry faeces) and urine (0.167 ng/ml) were used as reference samples. The significance of the difference between the mean of territorial and non-territorial males has been tested using a robust statistical test i.e. randomisation test. We prepared vectors for 1000 differences of randomised data (from real data). The 0.975 quantile (37.97) and normal 0.975 quantile (34.85) using the faecal testosterone were smaller than the difference between the mean of territorial and non-territorial males in real data (50.04). The 0.975 quantile (6.98) using the urine testosterone was slightly greater than the difference between territorial and non-territorial in real data (5.99), and the normal 0.975 quantile (5.94) approximated the difference between territorial and non-territorial in real data (5.99). Even though the sample sizes are small and hence our findings are somewhat equivocal, the testosterone data suggest that non-territorial resident males have an intermediate testosterone range i.e. lower than territorial males (significant for the faecal samples; t = 4.45, df = 3.07, p-value = 0.02; not significant for the urine samples, t = 1.56, df = 1.01, p-value = 0.36) but higher than transient males (significant for the faecal samples; t = 5.49, df = 2.70, p-value = 0.01; significant for the urine samples, t = 3.50, df = 2.78, p-value = 0.04) (Table 2). Levels of faecal testosterone were positively correlated with the frequencies of aggressive intra-sexual (Spearman rank correlation; r_s10_ = 0.7178, p = 0.0234) and of inter-sexual interactions (r_s10_ = 0.7915, p = 0.0088), the frequency of vocalization to addressed male targets and non-addressed (r_s10_ = 0.7316, p = 0.0202). The correlation between levels of testosterone and the frequency of vocalization to females (r_s10_ = 0.6074, p = 0.06) was inconclusive as the p-value approximate 0.05. Levels of faecal testosterone were negatively correlated with the frequencies of submissive intra-sexual interactions (r_s10_ = −0.9879, p<0.0001). We found no conclusive correlation between urine testosterone and intra- and inter-sexual interactions and pattern of vocalization.

**Table 2 pone-0012507-t002:** Descriptive statistics of testosterone levels in *A. forsteri* males displaying the territorial and non-territorial transient and resident mating tactics.

	Testosterone concentration							
	Territorial		Non-territorial		Transient		Resident	
	ng/g faeces	g/ml urine	ng/g faeces	g/ml urine	ng/g faeces	g/ml urine	ng/g faeces	g/ml urine
n	4	2	6	7	4	2	2	5
Mean	55.21	7.43	5.17	1.43	2.45	0.73	10.60	1.81
Median	47.75	*	3.65	1.58	1.60	*	*	1.74
SE	9.95	*	1.85	0.32	0.99	*	*	0.15
SD	19.91	*	4.53	0.72	1.99	*	*	0.33
Range (min-max)	40.70–84.65	3.89–10.96	1.20–11.70	0.51–2.38	1.20–5.40	0.51–0.96	9.50–11.70	1.58–2.38

*not relevant when n = 2.

### Parasitic load and species richness in faecal samples

**Figure 2:**
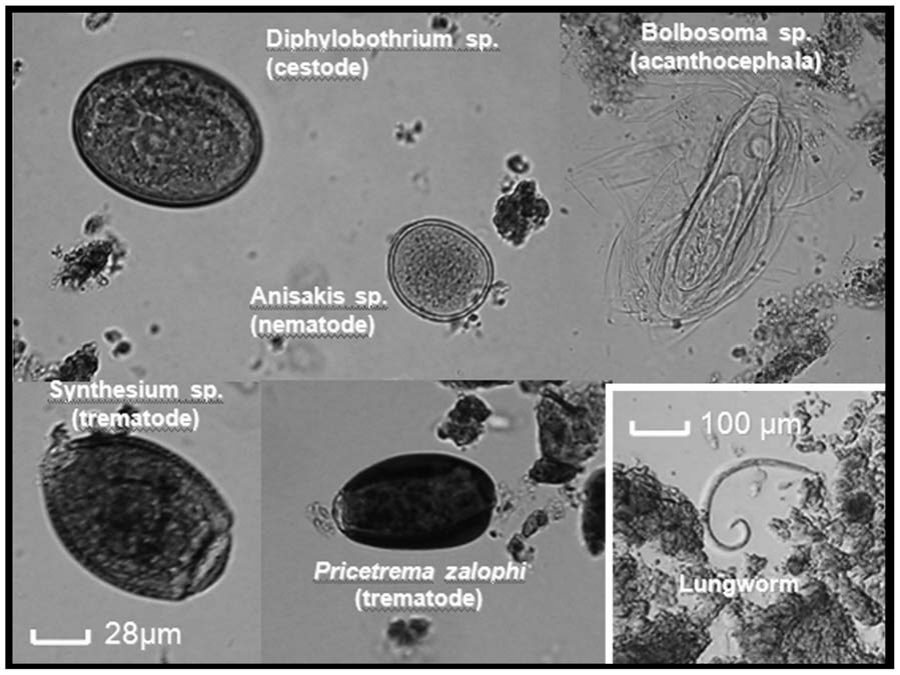
Common parasite species found in faecal samples collected from *A. forsteri* males.

A total of 12 faecal samples from different individual males were screened for parasite species (trematodes, cestodes, nematodes, acanthocephalans; Figure 2). We had testosterone measurements for 10 faecal samples out of 12. The parasite species diversity and descriptive statistics of parasite loads are detailed in Table 3. Two out of five territorial males had impressive numbers of eggs per gram of faeces (*Contracaecum* sp., 1575 and 2625 eggs/g of faeces; *Synthesium* sp., 2975 eggs/g of faeces; *Bolbosoma* sp., 1550 eggs/g of faeces). The original sites of parasite infection and the pathogenic characteristics of the observed parasite species described in previous studies are summarised in Table 4. Territorial males tended to be parasitised by a higher number of species compared to non-territorial males (Welch two sample *t*-test, t = 4.85, df = 6.62, p<0.002). The mean number of parasitic species per individual was 6.2±0.66 for the territorial males (n = 5) and 2.5±0.38 for the non-territorial males (n = 7).

**Table 3 pone-0012507-t003:** Descriptive statistics of parasite loads (eggs per gram of faeces) found in faeces of territorial and non-territorial males.

	Territorial			Non-territorial		
	n	median	Range	n	median	Range
**Cestodes**						
*Diphylobothrium*	4	187.5	125–500	5	125	75–500
**Nematodes**						
*Ascaris*	4	187.5	125–500	3	125	125–250
*Anisakis*	3	250	125–275	-	-	-
*Contracaecum*	3	1575	250–2625	2	100	100–100
*Ostostrongylus*	2	125	125–125	2	100	75–125
**Trematodes**						
*Synthesium*	3	500	125–2975	-	-	-
*Pricetrema*	1	50	50	1	100	100
*Zalophotrema*	1	125	125	-	-	-
**Acanthocephalans**						
*Bolbosoma*	3	375	125–1550	7	375	250–375
*Corynosoma*	1	50	50	-	-	-
UID 1	3	125	100–125	-	-	-
UID 2	3	75	75–125	-	-	-

The egg counts per gram of faeces in a female sample were used as reference sample and contained 500 *Diphylobothrium* and 100 *Ascaris* eggs/g. UID: unidentified.

**Table 4:** Parasite species found in faecal material of male New Zealand fur seals, original body part of parasite infection, and clinical signs reported in otariids and phocids.

*Parasite*	*Original site of infection*	*Clinical signs in otariids and phocids*	Notes
**Cestodes**			
*Diphylobothrium* spp.	GI	Pathogenic signs vary widely between hosts [57], [58].	Burdens may be high; infection vary seasonally.
**Nematodes**			
*Ascaris* spp.	GI	Gastritis, gastric ulceration, enteritis, diarrhea, dehydration, anemia, and gastric perforation [57], [59], [60].	Burden may be high with no apparent clinical signs.
*Anisakis* spp.	GI	As above	As above
*Contracaecum* spp.	GI	As above; Peritonitis and death induced by perforated ulcers in the proximal duodenum in California sea lions [61].	As above
*Ostostrongylus* spp.	RS	Vary widely between hosts; anorexia, depression, dehydration, neutrophilia, disseminated intravascular coagulation, death in elephant seals [62].	-
**Acanthocephala**			
*Bolbosoma* spp	GI	Death reported in Northern fur seal [58].	-
*Corynosoma* spp.	GI	As above	-
**Trematodes**			
*Synthesium* spp.	GI	ND	-
*Pricetrema* zalophi	GI	Colitis in infected elephant seals [63].	Burdens may be high with no apparent clinical signs
*Zalophotrema* spp.	Liver	Meningoencephalitis induced by aberrant trematode migration in California sea lions [64].	-

GI: gastrointestinal, RS: respiratory system, ND: no data available.

## Discussion

We recognise that this study is limited by small sample sizes, but given the system worked on, a marine mammal, the type and level of data we have been able to collect is quite exceptional and despite the small sizes the patterns observed are fairly robust and based on good statistical practice. Thus even while so constrained we believe that the overall pattern identified here, which holds strongly with evolutionary theory, will hold up to further investigation.

Immunosuppression by testosterone may be the result of resource allocation among activities with competing demands e.g. resources necessary for the maintenance of the immune functions may be reallocated to the production of costly secondary sexual characters, which have higher priority to increase mating success [9]. We considered that a mating tactic is a consequence of a combination of secondary sexual characteristics, hence we associate the non-territorial tactic with reduced secondary sexual characters and territorial tactic with enhanced secondary sexual characters.

Territorial *A. forsteri* males observed in this study showed more aggressive and sexual (dominant) behaviours compared to non-territorial males, in agreement with observations from earlier studies. In contrast, non-territorial males behaved in a subordinate way towards territorial males. In a synthesis derived from rodent, avian, and primate studies, Demas et al. [40] conclude that testosterone boosts reproductive effort by heightening sexual and aggressive behaviours, which in turn may provide advantages to male mating success. On the other hand, body size is an important facet of social rank in many mammals and particularly so in polygynous species where it directly influences the outcome of intra-sexual conflicts over access to females and consequently, male reproductive success [41], [42]. McGlothlin et al. [7] demonstrated positive correlations between the magnitude of testosterone concentration and the size of plumage ornament, which is an important determinant for female choice and male-male competition, in a population of dark-eyed juncos (*Junco hyemalis*). Female mate choice might also operate in *A. forsteri* where territorial dimorphic polygynous males can reach three times the size of adult females [43], [44]. Unfortunately, this study does not include any photogrammetry data and hence we are unable to investigate the association between intra-sexual size differences and testosterone levels directly. However, we did find that testosterone levels in territorial males were significantly higher than the testosterone levels in the non-territorial resident and transient males, further the few non-territorial resident males we sampled seemed to express higher testosterone concentrations compared to transient males. Social modulation of circulating hormone levels in males has been documented in a variety of species [45], [46], [47]. Sexual interactions or mere exposure to conspecific females increase sex hormone levels in a wide range of species [45]. Higher testosterone levels are generally encountered in territorial and high-ranking individuals across vertebrate taxa [14], [48], [49]. Non-territorial resident males spent a longer period of time in the study area compared to the transient males. Therefore, the resident males had a longer continuous exposure to receptive females and are expected to have higher testosterone levels than the transient males that had a shorter exposure to receptive females. Other studies in mammals and birds show positive effects of heightened testosterone on vocal displays and reproductive success [5], [14], [50]. We observed a higher mean frequency of vocalization to female and to male targets and of undirected vocalizations in territorial than in non-territorial males which we interpret as a higher degree of expression of secondary sexual characters. The main costs for a territorial otariid male might be related to the costs endured from male-male dispute and fights, to the permanent presence in its territory (maximal tenure estimated to 44 days in our study area), and to the depleted energy reserves from fasting during territory tenure rather then the vocal display itself. In *A. forsteri*, the occurrence of serious fights (e.g. biting resulting in deep wounds) is very low [44], which suggests that vocalizations could be one of the elements which regulate territorial conflict. The intra- and inter-sexual functions of vocalization might be to avoid costly confrontations since males might use vocal and visual cues to assess each other's current fighting ability and physical condition, while allowing females to assess a male's quality [51].

Caudron et al. [17] have shown that territorial *A. forsteri* males in the same study area tend to be larger and have higher reproductive success compared to non-territorial males. This work in conjunction with our own data suggests a strong positive, but unquantified, relationship between testosterone levels, territorial breeding tactic, size and reproductive success in our study males, thus the largest males, occupied the territorial positions, showed the highest levels of testosterone and were observed in more mating bouts. On the other hand, we found a positive relationship between male territoriality, testosterone levels and potentially parasitic loads. Note that all parasite species are not equally virulent, so that a larger number of parasite species in one host versus another does not necessarily equate with a greater impact of parasitism. In addition, the virulence of any particular parasite species is often dependent on the intensity of infection (Table 4). We found high parasite egg counts in some territorial males and based on Table 1–4, we can only speculate a positive association between dominance, high testosterone levels, parasitic infection, and its negative impacts on health.

Intra-sexual differences in parasite infections can be attributed to ecological (exposure) or physiological (susceptibility) causes. Ecological causes of such individual differences in parasitic infections include differential exposures to pathogens through diet, microhabitat choice and breeding behaviour [52]. The most plausible physiological (hormonal) explanations of such differences in parasitic infection are either the indirect effects of stress on the immune system, the direct effects of sex steroids on parasite growth and development, or the indirect effects of sex steroids on parasite establishment, growth, and development within the host through effect on immune system [52]. To our knowledge, no studies on the potential differences in diet between territorial and non-territorial male vertebrates are documented. Food-chain transmitted parasites are positively related to food consumption. Dominance status and body weight are often related and are indirectly a function of food consumption [53]. Consequently, somatic growth simultaneously increases an individual's probability of reaching high rank status and the potential for exposure to the food-chain transmitted parasites. *Arctocephalus forsteri* territorial males are larger and thus may have been feeding more intensively over the months preceding the breeding season. This increased feeding activity might have exposed them to an overall higher number of parasites and/or to more infective stages of the parasite species found in the faecal samples, because they are mainly acquired via food. In addition, *A. forsteri* males fast during breeding season, which may cause nutritional and physiological stresses placing an additional pressure on the immune functions and ultimately on health and survival in territorial males. One assumption of this study is that parasite burdens are independent of age. While Simokova et al. [54] show positive correlation between ectoparasite species richness and longevity among cyprinid fish species, studies in odd-toed hoofed mammals [55] show a negative correlation between parasite burdens and host longevity, while inconsistent patterns are observed among seabird species [56]. Non-territorial *A. forsteri* males include both sexually immature and mature males. Territorial status can be reached after both sexual and social maturity [43]. Furthmore, a male that is territorial during one breeding season might not hold territory in the following breeding season (Boren pers. communication).

In mammals, integrated studies on intra-sexual differences in behaviour, morphology, physiology, and parasite infections associated to the reproductive success of the different mating tactics are largely unexplored. A few studies examining the wild population of Soay sheep (*Ovis aries*) on the remote island of St Kilda have taken an integrated approach to this problem. The interactions between reproductive success, mating tactics, testosterone, and morphology have been examined [24], [25], while Coltman and collaborators [26] have looked at parasite burdens and their relationship to fitness in Soay sheep. Here, we provide valuable preliminary data suggestive of strong links between male mating tactics, testosterone levels and parasite loads, with potential effects on reproductive success and life history, as has been hypothesised based on life-history theory. This current study showed positive correlations between dominance, aggressive and sexual behaviour, testosterone levels, and potentially parasite loads. In addition, a joint study at the same study site showed a positive relationship between male territoriality, male body length, and reproductive success (four territorial males from reference 17 could be sampled for this study. Testosterone levels, reproductive efforts and parasite infections (in some territorial males) were higher in territorial males conferring these males with an increase in reproductive success, but may result in impaired health and ultimately lower survival in the long-term.
